# Supplementary material for: Tmem65 is critical for the structure and function of the intercalated discs in mouse hearts
Source: Nat Commun. 2022 Oct 18;13:6166. doi: 10.1038/s41467-022-33303-y (PMC9579145; doi:10.1038/s41467-022-33303-y)
Supplement: Supplementary file 1 — Supplementary Information [file 41467_2022_33303_MOESM1_ESM.pdf]

SUPPLEMENTARY FIGURE LEGEND

Figure S1. Reduced Tmem65 leads to a decrease in Cx43 transcript level and internalization of Cx43 proteins in the heart. (**a**) RT-qPCR showing Tmem65 (14.37±2.53% in Tmem65 KD hearts; 100±1.75% in control hearts) and Cx43 transcripts (30.32±5.73% in Tmem65 KD hearts; 100±2.75% in control hearts) in Tmem65 KD ventricles were significantly lower than control ventricles. ** $p < 0.01$. n=4. (**b**) Representative immunofluoresence showing Cx43 internalization in Tmem65 KD hearts. Cx43 was found on the ICD (yellow arrows) in control hearts, while some Cx43 was internalized (white arrows) in Tmem65 KD hearts. Cardiomyocytes were identified by cardiac sarcomeric α-actinin. Scale bar = 20 μm. Experiments were performed in mice of both sexes (n = 3 per group).

Figure S2. Tmem65 KD leads to prolonged PR and QRS intervals in the heart. (**a**) Representative surface ECG traces in Tmem65 KD and control hearts in awake animals. (**b**) A superimposition of ECG traces of scrambled control (black line) and Tmem65 KD (red line) hearts. ECG was performed in 10 control and 8 Tmem65 KD mice. Experiments were performed in mice of both sexes.

Figure S3. **Transmission electron microscopy showing aberrant mitochondria at the ICD in Tmem65 KD hearts**. Aberrant ICDs (right panel black arrow) and dismantled myofibrils (white arrow) were found in Tmem65 KD hearts. Only ICD-bound mitochondria in Tmem65 KD hearts were scattered (right panel), but sarcomeric mitochondria in Tmem65 KD heart (central panel, red arrows) were indifferent to control hearts (left panel, red arrows). Experiments were performed in mice of both sexes. 4 mice per group. (Scale bar = 500 nm).

Figure S4. Decreased mitochondrial contacts at the ICD of Tmem65 KD hearts. Mitochondria were loosely scattered at the ICD in Tmem65 KD hearts, but were densely packed in the same region of control hearts. **, $P<0.01$. Statistical analyses were performed by one-way ANOVA with Tukey's post-hoc test. Data were expressed as mean ± standard error of the means.

Figure S5. Immunofluorescence showing no change of sarcolemmal NaV1.5 in Tmem65 KD hearts. Representative image shows that no qualitative difference in sarcolemmal NaV1.5 (white arrows) between Tmem65 KD and control hearts. Scale bar = 20 μm. Experiments were performed in mice of both sexes.

Figure S6. Increased cardiac pacing at 10Hz led to aberrant Ca^{2+} transients in Tmem65 KD myocytes. Representative images of Ca^{2+} transients in control (left panel) and Tmem65 KD (right panel) cardiomyocytes paced at 10 Hz. Experiments were performed in mice of both sexes. 4 mice per group.

Supp. Figure 1

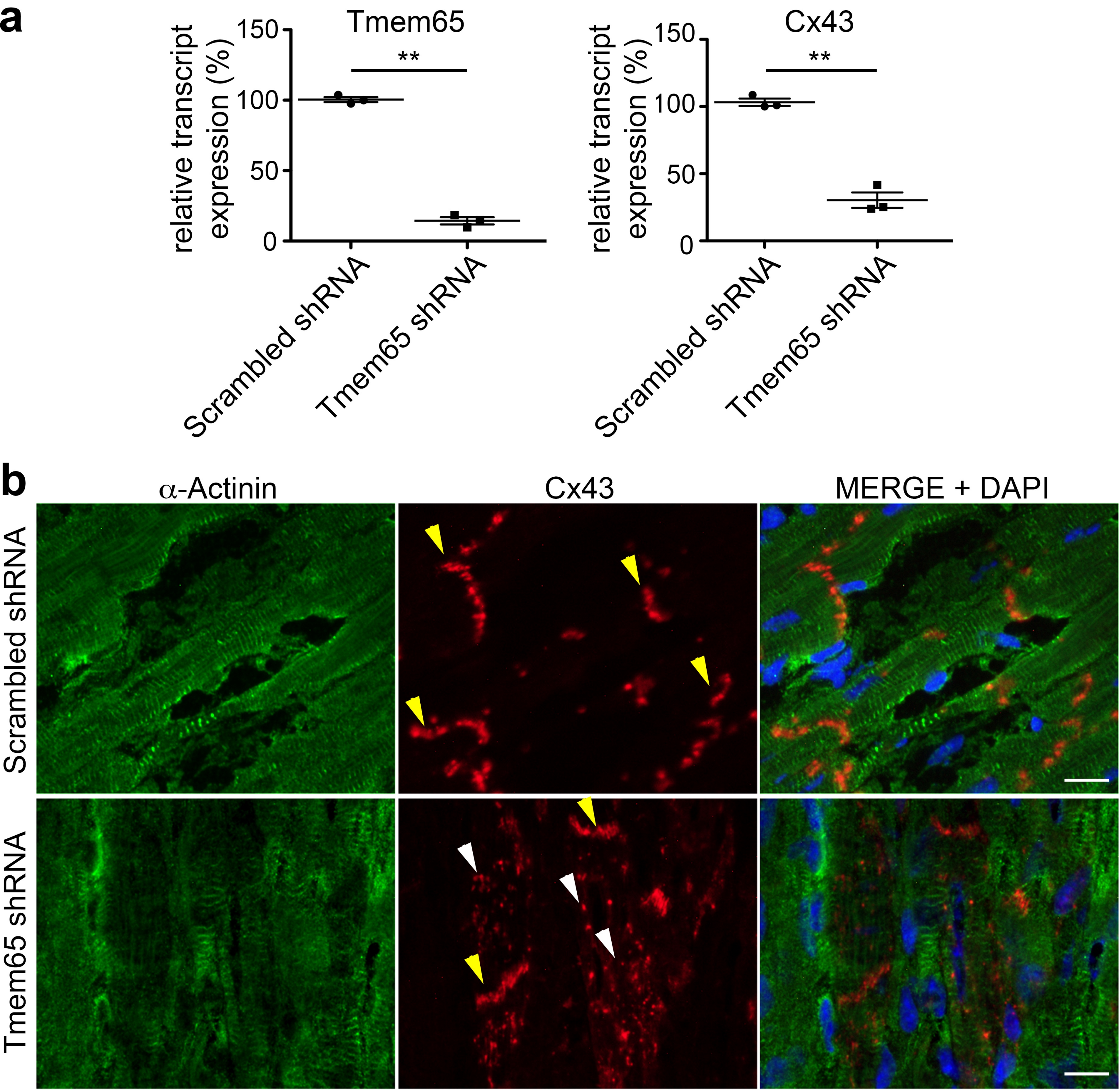

Supp. Figure 2

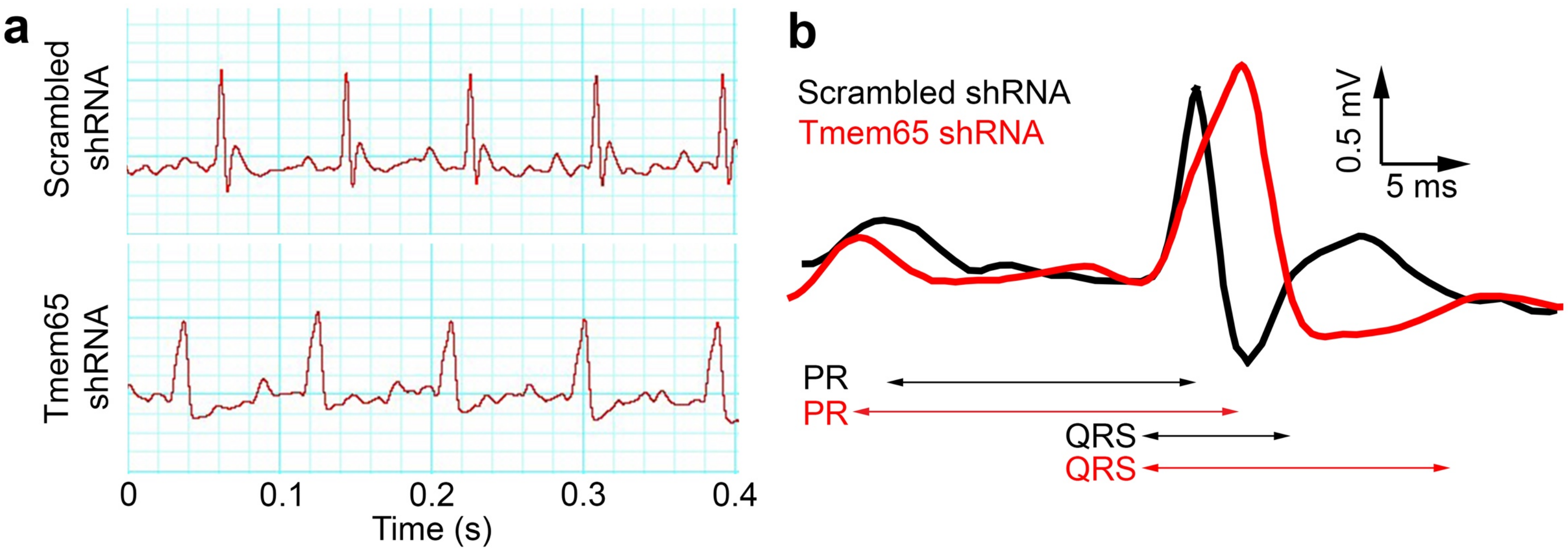

Supp. Figure 3

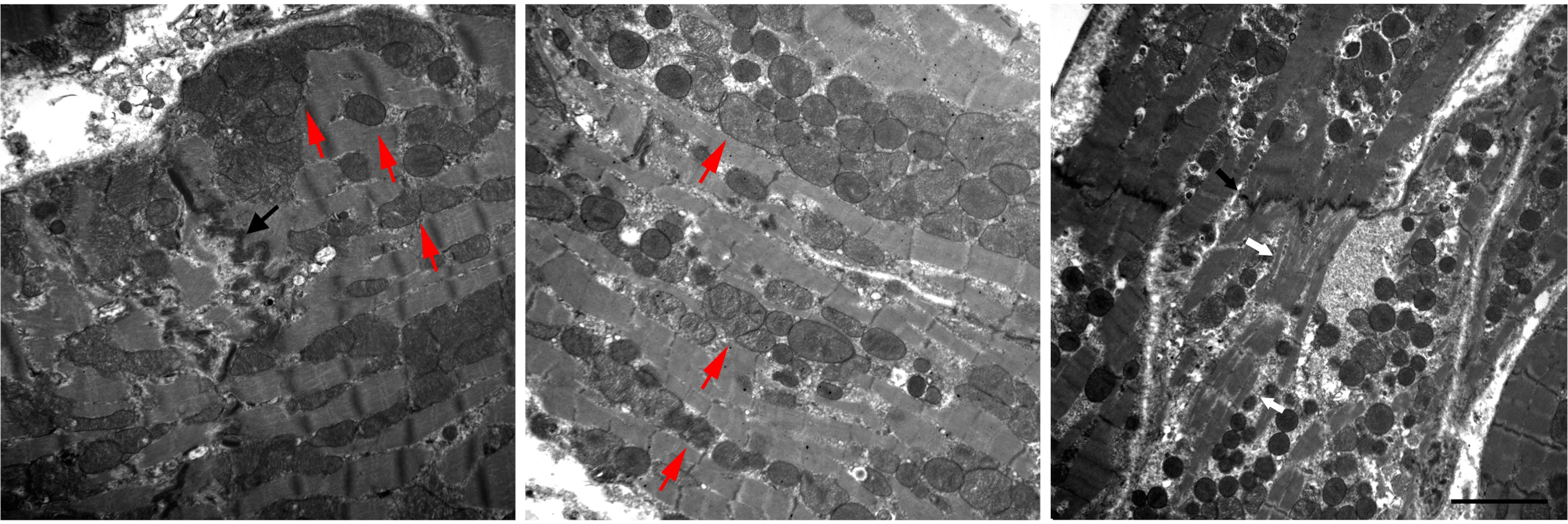

Scram. shRNA | Tmem65 shRNA | Tmem65 shRNA

Supp. Figure 4

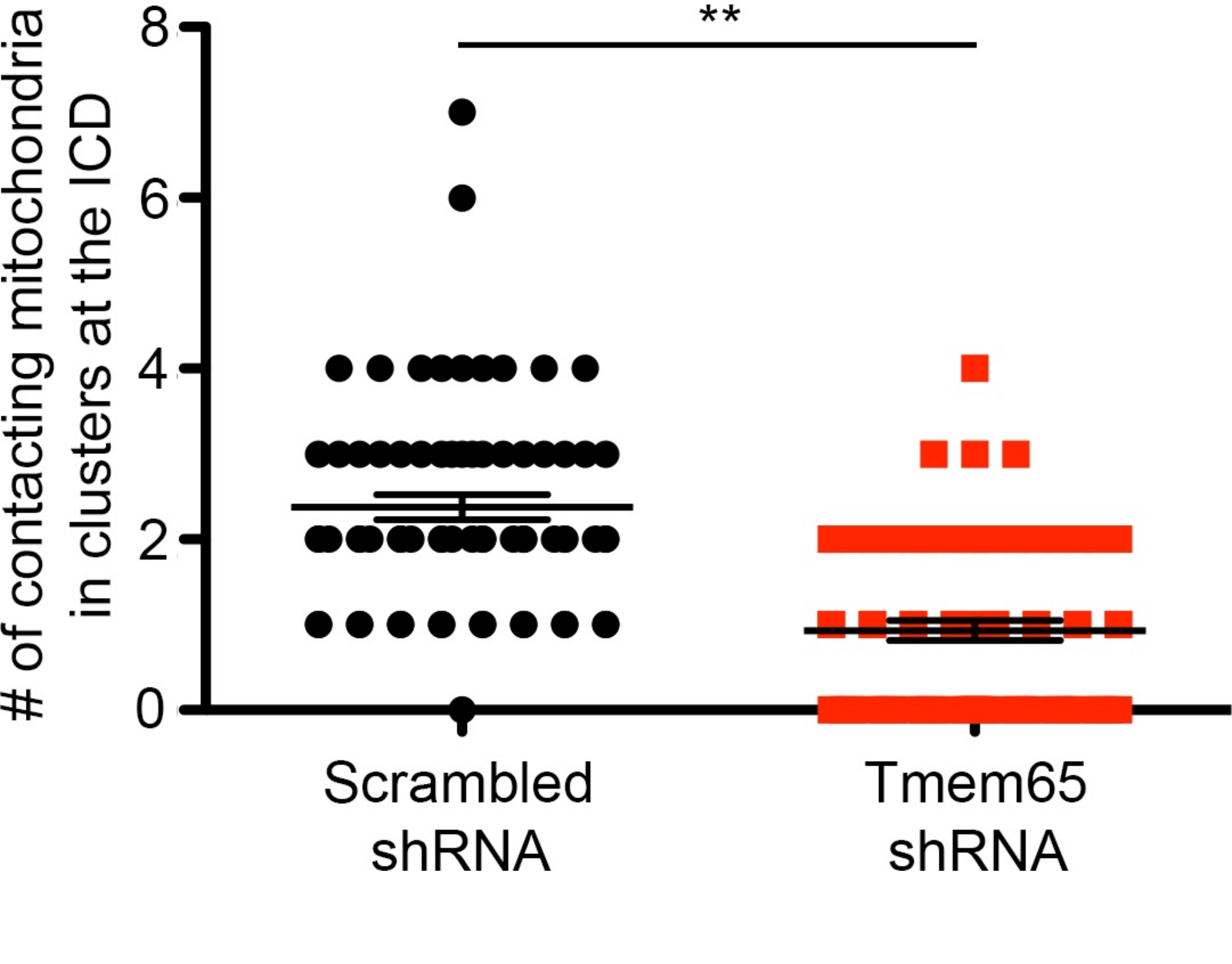

Supp. Figure 5

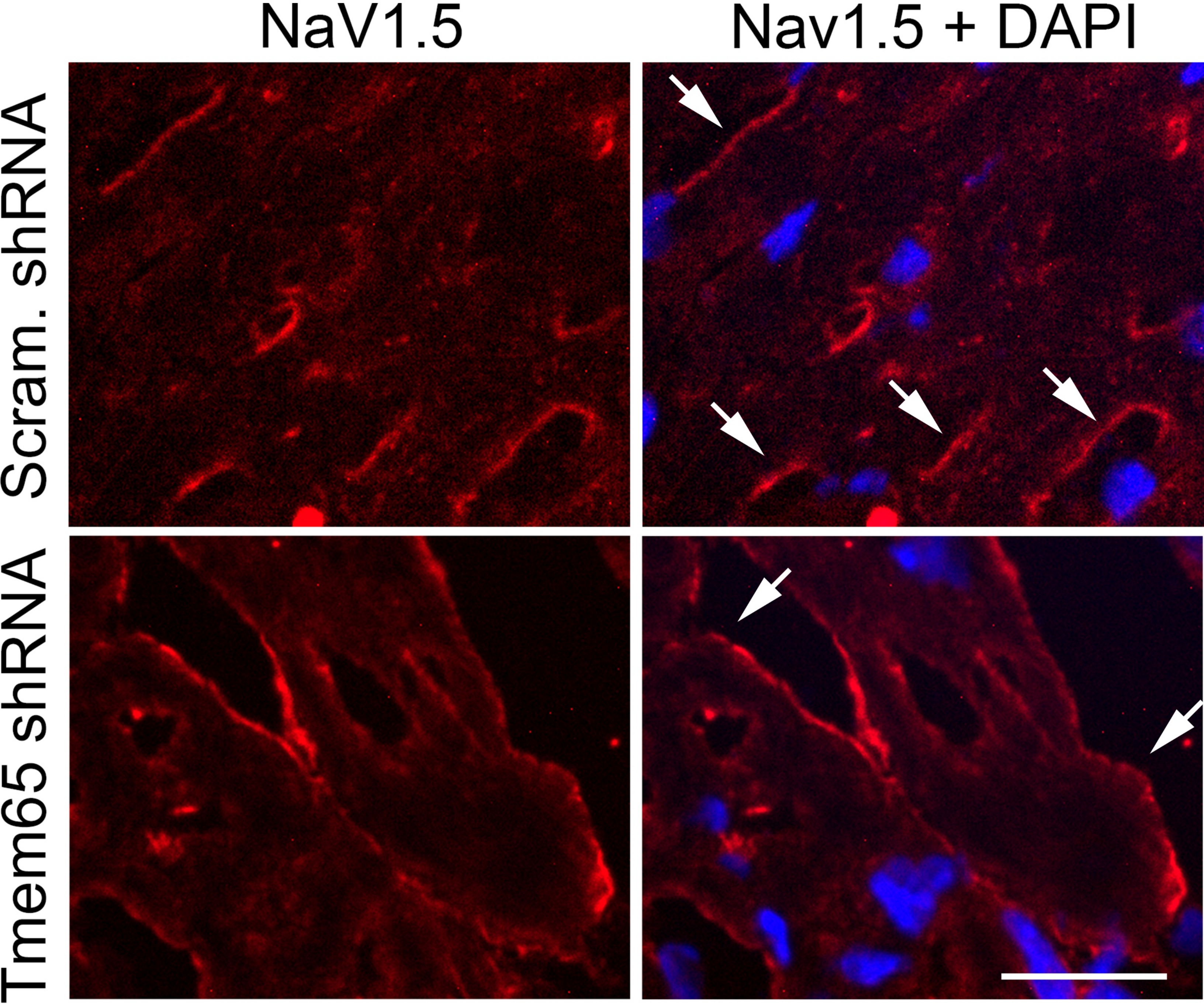

Supp. Figure 6

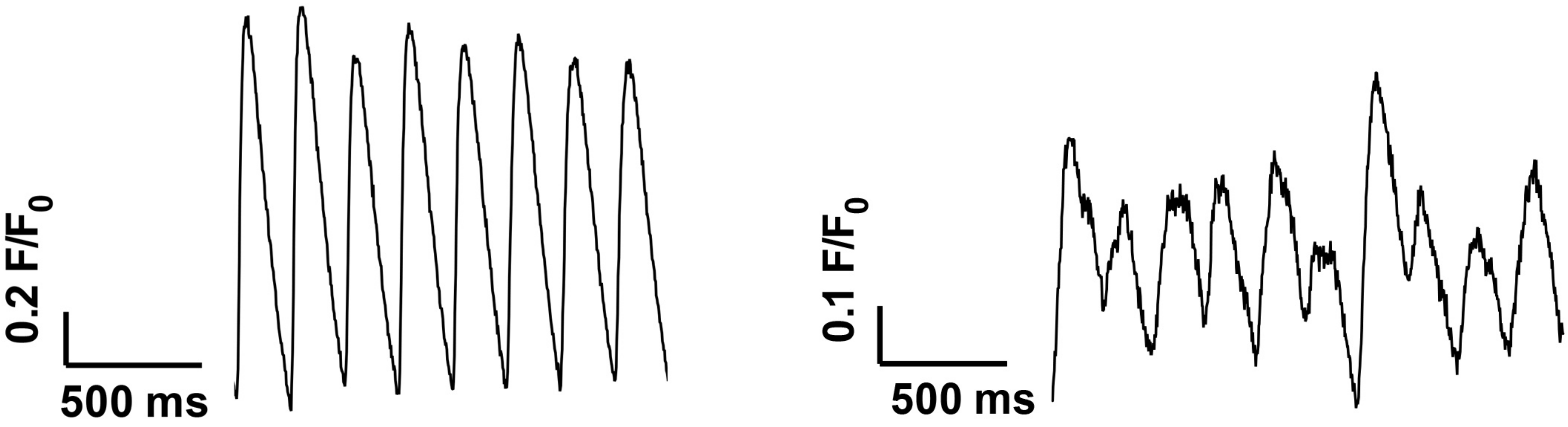

Scram. shRNA - 10 Hz   Tmem65 shRNA - 10 Hz
