# Supplementary material for: Tmem65 is critical for the structure and function of the intercalated discs in mouse hearts
Source: Nat Commun. 2022 Oct 18;13:6166. doi: 10.1038/s41467-022-33303-y (PMC9579145; doi:10.1038/s41467-022-33303-y)
Supplement: Supplementary file 3 — Description of Additional Supplementary Files [file 41467_2022_33303_MOESM3_ESM.pdf]

Description of Additional Supplementary Files

File Name: Supplementary Movie 1

Description: Scrambled shRNA mice.

File Name: Supplementary Movie 2

Description: Tmem65 shRNA mice.
