# Supplementary material for: Tmem65 is critical for the structure and function of the intercalated discs in mouse hearts
Source: Nat Commun. 2022 Oct 18;13:6166. doi: 10.1038/s41467-022-33303-y (PMC9579145; doi:10.1038/s41467-022-33303-y)
Supplement: Supplementary file 6 — Reporting Summary [file 41467_2022_33303_MOESM6_ESM.pdf]

nature research

Corresponding author(s): Anthony O. Gramolini
Last updated by author(s): June 22, 2022

Reporting Summary

Nature Research wishes to improve the reproducibility of the work that we publish. This form provides structure for consistency and transparency in reporting. For further information on Nature Research policies, see our Editorial Policies and the Editorial Policy Checklist.

Statistics

For all statistical analyses, confirm that the following items are present in the figure legend, table legend, main text, or Methods section.

n/a	Confirmed	
☐	☒	The exact sample size (*n*) for each experimental group/condition, given as a discrete number and unit of measurement
☐	☒	A statement on whether measurements were taken from distinct samples or whether the same sample was measured repeatedly
☐	☒	The statistical test(s) used AND whether they are one- or two-sided<br>*Only common tests should be described solely by name; describe more complex techniques in the Methods section.*
☒	☐	A description of all covariates tested
☐	☒	A description of any assumptions or corrections, such as tests of normality and adjustment for multiple comparisons
☐	☒	A full description of the statistical parameters including central tendency (e.g. means) or other basic estimates (e.g. regression coefficient) AND variation (e.g. standard deviation) or associated estimates of uncertainty (e.g. confidence intervals)
☐	☒	For null hypothesis testing, the test statistic (e.g. *F*, *t*, *r*) with confidence intervals, effect sizes, degrees of freedom and *P* value noted<br>*Give P values as exact values whenever suitable.*
☒	☐	For Bayesian analysis, information on the choice of priors and Markov chain Monte Carlo settings
☒	☐	For hierarchical and complex designs, identification of the appropriate level for tests and full reporting of outcomes
☐	☒	Estimates of effect sizes (e.g. Cohen's *d*, Pearson's *r*), indicating how they were calculated

Our web collection on statistics for biologists contains articles on many of the points above.

Software and code

Policy information about availability of computer code

Data collection	Zeiss Spinning Disc Confocal Microscope + Yokogawa CSU-X1 + Zeiss Zen Software (2012 S4) were used to acquire fluorescent and histology images.<br>JEOL 1400 Plus Transmission electron microscope was used to collect electron micrographs.<br>Olympus microscope (Modular IX83) + Prime BSI sCMOS camera were used to acquire super-resolution microscope images.<br>Bio-Rad ChemiDoc Imagers was used for acquiring immunoblot images.<br>**Complmentary metal oxide semiconductor camera (MiCAM Ultima-L, SciMedia) for dye transfer experiments in the mouse right ventricles.**<br>**Axopatch 200B voltage-clamp amplifier and pClamp 10.7 for patch clamp experiments**
Data analysis	Zeiss Zen Software for image merging and adult mouse cardiomyocyte dimension analysis.<br>ImageJ/Fiji/JACoP Plugin for co-localization study.<br>ImageJ/Fiji for protein densitometry and fibrosis analyses.<br>Echocardiography (Echo) was analyzed with Vevo 770 software, VisualSonic Inc., Toronto).<br>Electrocardiogram (ECG) was analyzed with EzCG Signal Analysis Software (Mouse Specifics, Inc).<br>**MATLAB-based ElectroMap software for determining conduction velocity in mouse hearts**

For manuscripts utilizing custom algorithms or software that are central to the research but not yet described in published literature, software must be made available to editors and reviewers. We strongly encourage code deposition in a community repository (e.g. GitHub). See the Nature Research guidelines for submitting code & software for further information.

Data

Policy information about availability of data

All manuscripts must include a data availability statement. This statement should provide the following information, where applicable:

- Accession codes, unique identifiers, or web links for publicly available datasets
- A list of figures that have associated raw data
- A description of any restrictions on data availability

A list of immunoblot raw data are provided as a supplement

Field-specific reporting

Please select the one below that is the best fit for your research. If you are not sure, read the appropriate sections before making your selection.

[x] Life sciences [ ] Behavioural & social sciences [ ] Ecological, evolutionary & environmental sciences

For a reference copy of the document with all sections, see nature.com/documents/nr-reporting-summary-flat.pdf

Life sciences study design

All studies must disclose on these points even when the disclosure is negative.

Sample size	For immunoblotting and immunofluorescent experiments, at least 4 mice from each group were chosen as minimal biological replicates. For ECG and EKG studies, at least 6 mice from each group were selected as minimal biological replicates. For transmission electron microscope, at least 3 mice from each group were included. Specific numbers of animals for each experiments are included in each figure legends and are determined to be statistically significant.
Data exclusions	Data were not excluded from analysis.
Replication	n = 3 replication was carried out to co-immunoprecipitation assays.
Randomization	Image acquisition was randomized for immunofluorescence.
Blinding	All experiments were carried out by at least 2 independent researchers for blinding.

Reporting for specific materials, systems and methods

We require information from authors about some types of materials, experimental systems and methods used in many studies. Here, indicate whether each material, system or method listed is relevant to your study. If you are not sure if a list item applies to your research, read the appropriate section before selecting a response.

Materials & experimental systems

n/a	Involved in the study	
[ ]	[x]	Antibodies
[ ]	[x]	Eukaryotic cell lines
[x]	[ ]	Palaeontology and archaeology
[ ]	[x]	Animals and other organisms
[x]	[ ]	Human research participants
[x]	[ ]	Clinical data
[x]	[ ]	Dual use research of concern

Methods

n/a	Involved in the study	
[x]	[ ]	ChIP-seq
[x]	[ ]	Flow cytometry
[x]	[ ]	MRI-based neuroimaging

Antibodies

Antibodies used

Immunoblots

Tmem65 antibody (1:1,000 dilution; Sigma, HPA025020), Connexin43 antibody (1:1,000; Sigma, C6219), α-Sarcomeric Actinin (1:1,000; Sigma, A7811), α-Sarcomeric Actin (1:1,000; Sigma, A2172), MyHC (1:40; DSHB,MF20), β-Actin (1:200; SC-47778, Santa Cruz Biotechology), cTnT (1:40; DSHB, CT3), MYH7 (1:40, DSHB, A4.840), alpha-Tubulin (1:40; DSHB, E7), FHL1 (1:1000; ab49241), Erk1/2 (1:1,000; Cell Signaling Technology, 9102), Phospho-Erk1/2 (1:1,000; Cell Signaling Technology, 9101), β1 (1:1,000, Cell Signaling Technology), NaV1.5 (1:1000; ASC-005, Alomone Labs), N-Cadherin (1:30 unconcentrated hybridoma supernatant; MNCD2, DSHB), FLAG (1:1,000; Sigma, F1804).

Immunofluorescence

Cx43 (1:100; C6219, Sigma), CoxIV (1:50, ab16056, Abcam), NCAD (1:20 unconcentrated hybridoma supernatant; MNCD2, DSHB), desmin (1:50 unconcentrated hybridoma supernatant; D3, DHSB), Alexa Fluor-568 phalloidin (A12380, Invitrogen, 150 nM),

	desmoplakin 1 and 2 (2722-5204, Bio-Rad, 1:100), and desmoglein (ab150372, Abcam, 1:250), NaV1.5 (1:500; ASC-005, Alomone Labs), NaV1.5 rabbit sera (1:50, in house) and purified β1 (1:500, in house).
Validation	Validation of commercially available antibodies are described on suppliers' websites and each antibody receives more than 10 citations for its biological purpose in the indicated application.<br><br>Validation and the use of Tmem65 (Sharma et al., 2015, Nat. Communs, PMID26403541), NaV1.5 rabbit sera and purified β1 (Veeraraghavan, 2018, Elife, PMID30106376) were previously reported.

Eukaryotic cell lines

Policy information about cell lines

Cell line source(s)	HEK293T cells (ATCC, CRL-3216)
Authentication	Authentication of cell lines is provided by ATCC.
Mycoplasma contamination	Mycoplasma contamination was tested by qPCR against the Mycoplasma genome.
Commonly misidentified lines (See ICLAC register)	*Name any commonly misidentified cell lines used in the study and provide a rationale for their use.*

Animals and other organisms

Policy information about studies involving animals; ARRIVE guidelines recommended for reporting animal research

Laboratory animals	CD-1 mice (Charles River Laboratory, Crl:CD1 (ICR))
Wild animals	Not applicable.
Field-collected samples	Not applicable.
Ethics oversight	All experiments involving animals were conducted in accordance with the Institutional Animal Care and Use Committee of the University of Toronto.

Note that full information on the approval of the study protocol must also be provided in the manuscript.
